# Subcortical structural variations associated with low socioeconomic status in adolescents

**DOI:** 10.1002/hbm.24796

**Published:** 2019-10-01

**Authors:** Lisanne M. Jenkins, Jessica J. Chiang, Katherine Vause, Lauren Hoffer, Kathryn Alpert, Todd B. Parrish, Lei Wang, Gregory E. Miller

**Affiliations:** ^1^ Department of Psychiatry and Behavioral Sciences Northwestern University Chicago Illinois; ^2^ Department of Psychology and Institute for Policy Research, Northwestern University Chicago Illinois; ^3^ Department of Radiology Northwestern University Chicago Illinois; ^4^ Department Biomedical Engineering Northwestern University Chicago Illinois

**Keywords:** adolescence, morphology, socioeconomic status

## Abstract

Low socioeconomic status (SES) is associated with a higher probability of multiple exposures (e.g., neighborhood violence, poor nutrition, housing instability, air pollution, and insensitive caregiving) known to affect structural development of subcortical brain regions that subserve threat and reward processing, however, few studies have examined the relationship between SES and such subcortical structures in adolescents. We examined SES variations in volume and surface morphometry of subcortical regions. The sample comprised 256 youth in eighth grade (mean age = 13.9 years), in whom high dimensional deformation mapping of structural 3T magnetic resonance imaging scans was performed. Vertex‐wise linear regression analyses examined associations between income to poverty ratio and surfaces of the hippocampus, amygdala, thalamus, caudate, putamen, nucleus accumbens and pallidum, with the covariates age, pubertal status, and intracranial volume. Given sex differences in pubertal development and subcortical maturation at this age, the analyses were stratified by sex. Among males, who at this age average an earlier pubertal stage than females, the relationship between SES and local shape variation in subcortical regions was almost entirely positive. For females, the relationship between SES and local shape variation was negative. Racial identity was associated with SES in our sample, however supplementary analyses indicated that most of the associations between SES and subcortical structure were independent of it. Although these cross‐sectional results are not definitive, they are consistent with a scenario where low SES delays structural maturation of subcortical regions involved with threat and reward processing. Future longitudinal studies are needed to test this hypothesis.

## 1 INTRODUCTION

Compared with more affluent peers, children from families of low socioeconomic status (SES) have disproportionately worse outcomes in multiple domains. These include cognitive development and language acquisition (Noble, McCandliss, & Farah, 2007), executive control (Raver, Blair, & Willoughby, 2013) and school achievement (Duncan & Murname, 2011). Lower SES youth also experience higher rates of some mental and physical health problems, including depressive symptoms, externalizing disorders (Reiss, 2013), obesity (Wang & Beydoun, 2007), insulin resistance (Goodman, McEwen, Huang, Dolan, & Adler, 2005), high blood pressure (Chen, Matthews, & Boyce, 2002), and early atherosclerosis (Matthews & Gallo, 2011). Many of these risks persist across the lifecourse (Hertzman & Boyce, 2010). In adulthood, low‐SES children have higher risk for mood disorders, cardiovascular disease, and premature mortality (Green et al., 2010; Miller, Chen, & Parker, 2011).

Understanding why children from low‐SES families show vulnerability to a heterogeneous set of adverse life outcomes is challenging. Low SES is associated with a higher probability of multiple exposures (e.g., neighborhood violence, poor nutrition, housing instability, air pollution, unfair treatment, and insensitive caregiving) known to affect structural development of subcortical brain regions that subserve threat and reward processing, including the hippocampus, amygdala, basal ganglia, and thalamus (Gianaros & Hackman, 2013; Nusslock & Miller, 2016; Sheridan & McLaughlin, 2014). Although these subcortical regions are also crucial for numerous other functions, including memory, attention, and learning (Packard & Knowlton, 2002; Portas et al., 1998; Squire, 1992), their importance in threat and reward processing may be related to exaggerated threat reactivity in youths from low‐SES backgrounds, which manifests in greater sympathetic, hormonal, and inflammatory responses to stressors, and altered reward processing, contributing to dysphoria, substance use, and disinhibited eating. Collectively, these processes theoretically accentuate vulnerability to mental and physical health problems (Nusslock & Miller, 2016). Evidence comes from findings that volumes of subcortical regions, in particular the amygdala, are associated with threat sensitivity (Carlson et al., 2012; Foell et al., 2019). Heightened sensitivity to threat and reduced sensitivity to reward have also been associated with anxiety and depression, respectively (Shankman et al., 2013). Volumes of subcortical regions involved in threat and reward processing have also been implicated in mood disorders, for example, decreased hippocampus and thalamus volumes in bipolar disorder (Hibar et al., 2016) and basal ganglia in major depressive disorder (Lorenzetti, Allen, Fornito, & Yucel, 2009). Children who have experienced physical abuse and children from low‐SES families both have reductions in amygdala and hippocampal volume (Hanson et al., 2015).

Multiple studies have found associations between SES and subcortical brain structures in youths, particularly in threat‐processing regions. Lower family SES has generally been linked to smaller hippocampal volume in children and adolescents (Hanson et al., 2015; Hanson, Chandra, Wolfe, & Pollak, 2011; Jednorog et al., 2012; Leonard, Mackey, Finn, & Gabrieli, 2015; Luby et al., 2013; McDermott et al., 2019; Noble, Houston, Kan, & Sowell, 2012; Yu et al., 2017). Findings for the amygdala have been mixed, with links to smaller amygdala volumes reported in some cohorts (Hanson et al., 2015; Luby et al., 2013; McDermott et al., 2019), but not others (Hair, Hanson, Wolfe, & Pollak, 2015; Hanson et al., 2011; Noble et al., 2012, 2015). Few studies have examined SES variations in the structure of subcortical reward‐related regions. Leonard et al. (2015) observed that adolescents from low‐SES backgrounds had smaller caudate volume compared to their high‐SES counterparts; however, this difference was not statistically significant, perhaps due to the relatively small sample (*N* = 58). A larger study of 325 youth (Ball et al., 2012) also did not find associations between SES and volume of the basal ganglia or thalamus; however, stringent health‐based eligibility criteria may have obscured associations, as more recently, McDermott et al. (2019) found that low SES was associated with smaller volume of the striatum and thalamus.

The present study attempted to clarify the relationship between SES and subcortical structures in a large, inclusive sample of youths. Previous studies have used regional volume estimates to quantify brain structure. These coarse estimates obscure local variations in shape, and may yield false negative findings by aggregating across local regions. Here, we localize SES effects using three‐dimensional surface analyses. This approach can reveal subtle disparities in regional shape with precision that is not apparent on standard volumetric analysis (Mamah, Barch, & Csernansky, 2009), and has been used previously to identify focal morphological effects of SES on subcortical brain regions in individuals aged between 5 and 25 years (McDermott et al., 2019). In previous research (Csernansky, Wang, Joshi, Ratnanather, & Miller, 2004), we have established that diffeomorphic mapping of structural magnetic resonance imaging (MRI) resulted in reliable and valid maps between anatomical atlases and participant scans, with sub‐mm precision. Furthermore, we have demonstrated valid and reliable surface‐based representations of anatomical structures based on these maps, which produced disease‐specific biomarkers of morphological patterns (Csernansky et al., 2004; Wang et al., 2006, 2007).

We considered these questions here in a cross‐sectional, economically diverse sample of urban youth. Because it is difficult to parse environmental from maturational influences, youth were studied during a common developmental period, the eighth grade. Studies report differences in subcortical brain volumes between males and female adolescents of the same age (Giedd et al., 2015; Uematsu et al., 2012). These sex‐specific changes in subcortical brain structure are posited to be related to puberty (Wierenga et al., 2018), and are nonlinear (Giedd et al., 2015; Vijayakumar, Op de Macks, Shirtcliff, & Pfeifer, 2018), potentially accounting for heterogeneous findings (Dennison et al., 2013; Wierenga et al., 2014). Furthermore, chronic stress has been shown to produce sex‐specific morphological changes in subcortical brain structures (McLaughlin, Baran, & Conrad, 2009). Therefore, we elected to examine how SES relates to structural indices separately in males and females. We expected that male and female youths would show distinct associations between SES and subcortical structure. Specifically, we hypothesized that lower SES would covary with smaller regional volume and more inward (concave relative to the population average) local shape variation of regions involved with threat (amygdala, hippocampus, and thalamus) and reward (caudate, putamen, pallidum, and nucleus accumbens), and that males and females would show these associations in distinct local regions of these structures.

## 2 MATERIALS AND METHODS

### 2.1 Participants and procedures

Participants were 277 youth from the greater Chicago community. All youths were in the eighth grade, with an average age of 14 years (range = 11.83–15.33). Youth and a parent were screened for eligibility via telephone. Exclusion criteria were: history of chronic medical or psychiatric illness, infectious disease during the past 2 weeks, taking prescription medication during the prior 3 months, and being currently pregnant. Youths and parents provided written informed consent prior to participating, and the study was approved by the Institutional Review Board at Northwestern University. Eligible youth attended a laboratory visit, during which the parent completed interviews about their SES and youth completed a series of psychosocial questionnaires and behavioral tasks. On a separate day, youth completed an MRI scan at Northwestern University's Center for Translational Imaging.

The authors assert that all procedures contributing to this work comply with the ethical standards of the relevant national and institutional committees on human experimentation and with the Helsinki Declaration of 1975, as revised in 2008.

Of the 277 youth enrolled, 15 were not scanned because they could not schedule, or were too obese or anxious for the scanner. Following visual inspection (see below), five individuals were excluded due to poor segmentations in one or more structures (three female, two male), and one due to agenesis of the corpus callosum (one female). Thus, the final analytic sample was 256. Demographic information is displayed in Table 1. As expected, compared to male youth, female youth had significantly smaller intracranial volume and were at a more advanced pubertal stage. Age was not significantly correlated with intracranial volume (ICV) or income to poverty ratio (IPR), but was significantly correlated with pubertal stage (rho = .261, *p* < .0001). IPR was significantly correlated with ICV (*r* = .278, *p* < .00001), more strongly in males (*r* = .306, *p* = .002) than females (*r* = .211, *p* = .007), and was not correlated with pubertal stage. Pubertal stage (higher score = more advanced) was negatively associated with ICV (rho = −.256, *p* < .0001) in both males and females, but positively associated with ICV in males only (rho = .222, *p* = .03) and n.s. in females only.

**Table 1:** Demographic description of the sample

	Male (*n* = 96)	Female (*n* = 160)	Statistics
	Mean (*SD*) or *n*	Mean (*SD*) or *n*	
Age in years	13.90 (0.49)	13.92 (0.56)	*t*(254) = 0.40, *p* = .691
Caucasian (*n*)	Yes = 42, no = 54	Yes = 62, no = 98	*χ* ^2^ (1) = 0.62, *p* = .43
Hispanic (*n*)	Yes = 32, no = 64	Yes = 48, no = 112	*χ* ^2^ (1) = 0.31, *p* = .58
Puberty category (*n*)	Prepubertal = 1	Prepubertal = 0	*χ* ^2^ (4) = 98.65, *p* < .001
	Early pubertal = 10	Early pubertal = 0	
	Mid‐pubertal = 59	Mid‐pubertal = 21	
	Late‐pubertal = 24	Late‐pubertal = 104	
	Postpubertal = 2	Postpubertal = 35	
Intracranial volume (cm^3^)	1,619.10 (134.74)	1,464.73 (120.91)	*t*(254) = −9.47, *p* < .001
Income: Poverty ratio winsorized	4.14 (4.11)	3.33 (2.81)	*t*(148.59) = −1.72, *p* = .088

### 2.2 Socioeconomic status

As with past research (Luby et al., 2013; Noble et al., 2012; Whittle et al., 2017), we defined SES by household's IPR. Parents or guardians reported all sources of household income (wages, social security, disability, and unemployment benefits, worker's compensation, inheritances, and help from relatives) during the previous calendar year and composition of the household (including ages of any children). This information, along with the federal government's poverty threshold for the calendar year prior to study entry was used to compute a continuous measure of IPR. Higher IPR values reflect greater material resources. Three participants (1.2%) had extreme IPR values (>3 *SD*s), which were winsorized with the next largest value (17.27) in the sample. An IPR value below 1 is classified as poverty (Kim, Ho, Evans, Liberzon, & Swain, 2015), thus 19% of males and 17% of females in this study were living in poverty according to this U.S. Census Bureau calculation. Another 20% of males and 22% of females had an IPR value of 1.00–1.99, a range that is commonly designated as low income.

### 2.3 Image acquisition and surface mapping

Structural scans were acquired using T1‐weighted Magnetization prepared rapid gradient echo imaging (TR = 2,300, TE = 1.91, voxel size = 0.80 isotropic, FOV = 320x320, and flip angle = 7). Head motion was controlled in the scanner by foam or inflatable padding and conveying the importance of staying still to participants. Surfaces of the subcortical structures of the hippocampus, amygdala, basal ganglia (caudate, putamen, pallidum, and nucleus accumbens) and thalamus were automatically generated for each participant using the FS + LDDMM pipeline (Khan, Wang, & Beg, 2008). This technique combines FreeSurfer's (FS) probabilistic voxel‐based classification (Desikan et al., 2006) and a deformable, high‐dimensional template‐based method of large diffeomorphic metric mapping, (LDDMM; Beg, Miller, Trouve, & Younes, 2005). FS (version 5.3) subcortical labeling (Fischl et al., 2002) obtained the initial subcortical segmentations. This was followed with image alignment and intensity normalization with LDDMM (Beg et al., 2005), which produces smooth transformations for each region of interest (ROI). Visual quality control of FSLDDMM outputs overlaid on each T1 image was performed to exclude visible segmentation errors of the seven subcortical ROIs under study. This included taking into account the overall quality of the T1 image, including amount of ghosting, it is contrast and blurriness due to movement as well as the object map's ability to fully encompass the outline of the ROI.

### 2.4 Subcortical surface processing

Subcortical surfaces for each participant were rigidly registered to atlas space to calculate a population average (Csernansky et al., 2004). For each participant, local shape variation was calculated from the population average of all participants by quantifying the perpendicular amplitude between surfaces at a vertex‐to‐vertex level. The quantification of perpendicular change between surfaces was assigned a positive (outward variation [convex] from population average) or negative (inward variation [concave] from population average) sign. For each ROI, shape variation values at each vertex were summed across the whole surface. Overall volume for each participant for each ROI was calculated utilizing the volume enclosed within the surfaces. Mean subcortical volumes for males and females are reported in Table S1.

### 2.5 Covariates and supplemental analyses

Age, pubertal status (from the Pubertal Development Scale; Petersen, Crockett, Richards, & Boxer, 1988) and total ICV estimated by FS segmentation were included as covariates in surface analyses, as these factors have been shown to influence subcortical volumes (Herting et al., 2014; Lenroot et al., 2007; Potvin, Mouiha, Dieumegarde, & Duchesne, 2016). In supplementary analyses, we also performed the surface analyses without ICV as a covariate (Figure S1) as well as with children's self‐described racial (Caucasian Y/N) and ethnic (Hispanic Y/N) identities as covariates (Figure S2). Volume estimates of ROIs are reported in the Supplement (Table S1) along with linear regressions for these (Tables S2 and S3), for the interested reader.

### 2.6 Experimental design and statistical analyses

This was a cross‐sectional study. Associations between IPR and local shape variation of each subcortical region were examined in surface‐based analyses performed using SurfStat (Chung, Worsley, Nacewicz, Dalton, & Davidson, 2010) implemented in MATLAB. Morphometric values were regressed onto IPR scores to localize significant regions of shape variation. Separate models were tested for each ROI, and age, puberty status, and ICV were covaried in all models, except in Supplementary analyses, described above.

To account for multiple comparisons, random field theory (RFT; Taylor & Worsley, 2007; Worsley, Andermann, Koulis, MacDonald, & Evans, 1999) was applied using SurfStat. RFT considers both peaks and spatial extent by modeling noise as Gaussian random fields. This approach provided significant clusters of vertices at the family‐wise error rate (FWER) of *p* < .01 and a FWER of *p* < .05 within each ROI. Bonferroni correction was used to correct for multiple comparisons across ROIs (*p* = .05/7 ROIs) resulting in *p* < .007, corrected for each ROI. Significance was visualized as a color map on the overall average surface.

## 3 RESULTS

### 3.1 Subcortical local shape variation

**Figure 1:**
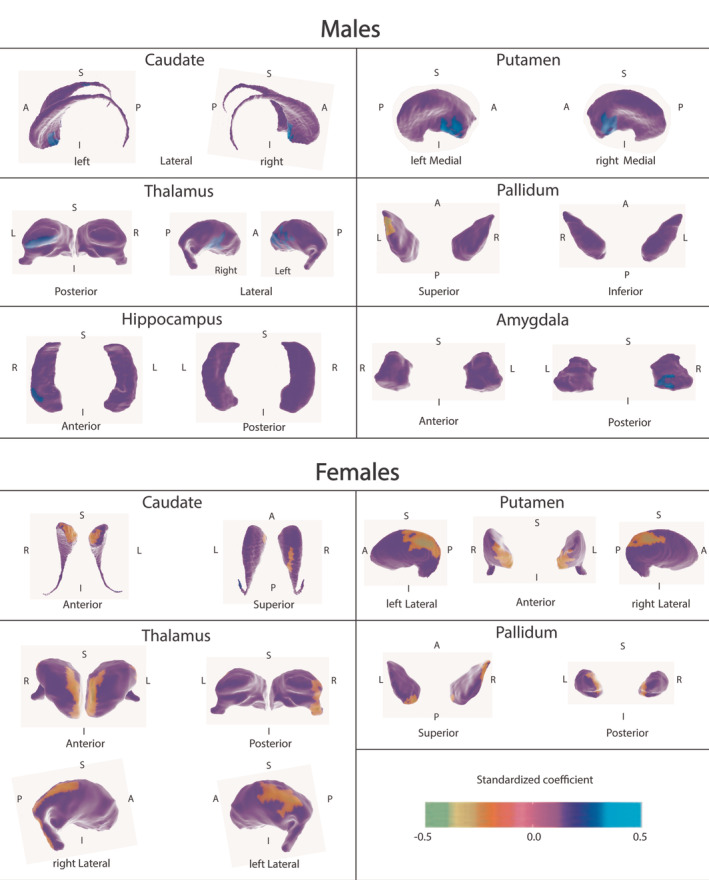
Regression of local shape variation onto Income: Poverty ratio for males and females, with age, puberty category, and intracranial volume as covariates. In regions with warmer colors, lower IPR is related to more outward local shape variation. Vertex‐wise RFT corrected FWER cluster threshold *p* < .01 within each ROI, FWER *p* < .007 per ROI, Bonferroni corrected for multiple comparisons. In regions with cooler colors, lower IPR is related to more inward local shape variation. *Note*: A, anterior; FWER, family‐wise error rate; I, inferior; IPR, Income: Poverty ratio; L, left; P, posterior; RFT, random field theory; R, right; ROI, region of interest; S, superior. Figure S1 shows these structures in their correct anatomical relationship with each other, as they appear *in situ*

Figure 1 shows the results of the shape analysis. Regions in green do not have significant associations with IPR. Warm colors indicate regions where IPR is positively associated with local shape variation (lower IPR is related to more outward local shape variation) and cool colors indicate areas where it is negatively associated with local shape variation (lower IPR is related to more inward local shape variation).

For males, the general pattern across structures was a positive association, reflecting the covariation of lower IPR with outward local shape variation. Figure 1 shows that for males, lower IPR was associated with outward local shape variation of the lateral caudate head, lateral thalamus and medial putamen bilaterally, and the left posterior thalamus, right posterior amygdala, and right anterior hippocampus. By contrast, associations in the pallidum were negative, such that lower IPR covaried with inward local shape variation of the left superior pallidum.

For females, lower IPR covaried with inward local shape variation of the bilateral anterior caudate head, anterior and postero‐lateral putamen, supero‐medial and postero‐lateral thalamus, and postero‐medial pallidum, as well as the right superior caudate body, posterior thalamus, and antero‐superior pallidum. IPR was also positively associated with a cluster of outward local shape variation in the left caudate tail.

#### 3.1.1 Racial and ethnic identity as additional covariates

In the United States, SES varies by racial and ethnic group, and we observed IPR differences in our sample (Caucasian = 6.19 [*SD* = 4.10], African American = 2.29 [*SD* = 2.01], Latino = 2.43 [*SD* = 1.79], *F*[2, 253] = 52.14, *p* < .001). Therefore, in supplemental surface analyses we estimated the models with additional covariates reflecting children's self‐described racial (Caucasian Y/N) and ethnic (Hispanic Y/N) identities. Inclusion of these covariates had very little effect on the magnitude of the observed relationships between IPR and surface variation in males (Figure S3). These covariates attenuated the magnitude of some associations in females (Figure S4).

#### 3.1.2 Volume

As most literature in this area reports volume rather than surface analyses, for sake of comparison we present subcortical volumes in the Supplement (Table S1). Multiple regression analyses for males (Table S2), controlling for age, puberty, and ICV, found that IPR was not significantly associated with volume of any structure after Bonferroni correction for multiple comparisons (*p* < .007). For females, controlling for age, puberty, and ICV, IPR was significantly associated with volume of the caudate, pallidum, putamen, and thalamus, after Bonferroni correction.

## 4 DISCUSSION

In a relatively large, diverse sample of youths, we examined the association between IPR and the structure of subcortical regions, using a sex‐stratified, surface‐analysis approach. As hypothesized, males and females showed associations between IPR and brain morphology in different local regions. Unexpectedly, IPR was positively associated with subcortical shape variation in males but negatively associated with shape variation in females. A similar pattern was observed for volume (see Supporting Information). To understand these findings, we suggest it is useful to consider the normative sex differences in pubertal development and brain maturation among eighth graders (as a reminder, in this sample females were markedly more advanced than males in terms of puberty, *p* < .001, but n.s. in age). As children transition into adolescence, gray matter decreases due to synaptic pruning, and white matter increases due to myelination, changes which serve to improve computational efficiency (Giedd et al., 2015). These maturational changes affect volume and the gray matter changes are also not often linear, with disparate trajectories in males and females (Giedd et al., 2015; Uematsu et al., 2012). In our study, IPR was correlated with ICV. ICV was negatively correlated with pubertal stage when males and females were considered together, but positively correlated when only examining males. Given these findings and those from previous longitudinal studies (Giedd et al., 2015; Uematsu et al., 2012), the males in our sample are likely to have been scanned at a pubertal stage before they had reached peak volume of subcortical structures. As a result, they were likely to be on an upward maturational trajectory with regard to volume. By contrast, females in the study are likely to have been scanned after they had passed the pubertal stage of peak subcortical volume, as identified by longitudinal studies examining age (Giedd et al., 2015; Uematsu et al., 2012). As a consequence, they are likely to have been on a downward maturational trajectory of volume. To the extent these observations are accurate, they suggest that higher SES is associated with faster maturation of the subcortical regions identified. The direction of our findings differ by sex simply because at the pubertal stage measured, subcortical volume was still generally increasing among boys, while it was generally decreasing in girls.

Of course, in a cross‐sectional design like this, we cannot substantiate this interpretation; longitudinal data across childhood would be needed. To our knowledge, there is only a single published study of childhood SES that separately considered males and females (Lawson et al., 2017). It found that lower childhood SES was associated with smaller hippocampal volume in adult women (25–36 years old), but larger hippocampal volume in adult men, although not quite to a significant extent in a small sample. Nevertheless, this result is somewhat consistent with our findings, in that we found that lower SES was associated with outward hippocampal curvature in males.

Regions that are important for the regulation of social behavior, including subcortical regions such as the hippocampus, basal ganglia, and amygdala, undergo protracted development which renders them vulnerable to experience‐dependent changes influenced by the environment for prolonged time periods (Miskolczi, Halasz, & Mikics, 2019). These time epochs of adaptive and dynamic brain changes are known as sensitive periods, and are regulated in part by brain‐derived neurotrophic factor (BDNF; Miskolczi et al., 2019). BDNF reaches peak levels in adolescence and is involved in regulation of programmed cell death, synaptic pruning, and axonal and dendritic branching of rapidly proliferating and differentiating neurons (Yang et al., 2009). Early life social adversity results in abnormalities in BDNF methylation and mRNA expression, including in the hippocampus and amygdala (Miskolczi et al., 2019). Also, there are sex differences in BDNF expression (Bath, Schilit, & Lee, 2013) and sensitive periods, and associated effects on amygdala and hippocampal volume (Pechtel, Lyons‐Ruth, Anderson, & Teicher, 2014; Teicher et al., 2018). In their model, Miskolczi et al. (2019) argue that sex (along with genetic factors) influence brain developmental curves, that the trajectory of these developmental curves are region‐specific and furthermore determine the timing of sensitive periods for each region, and that depending on the timing of the early life adversity, separable brain regions might undergo disturbed development, resulting in aberrant maturation and increased risk of psychopathology.

### 4.1 Region‐specific patterns

Few studies have examined associations between SES and basal ganglia structure (Ball et al., 2012; Leonard et al., 2015; McDermott et al., 2019). We found that in males, low IPR was associated with outward curvature of basal ganglia regions including the lateral caudate head and antero‐medial putamen. In females, low IPR was associated with inward curvature of the bilateral caudate head, right superior caudate body, bilateral anterior and poster‐lateral putamen. Low IPR was also associated with inward curvature of the left and bilateral pallidum in males and females, respectively. Supplemental analysis of volume found that IPR was also negatively associated with volume of the caudate, putamen, and pallidum in females. These findings contrast those of Ball et al. (2012) who did not find that SES was associated with reduced basal ganglia volume, and are consistent with Leonard et al. (2015) who found reduced caudate volume in adolescents from low SES backgrounds, albeit nonsignificant. Our significant basal ganglia findings also contrast those of McDermott et al. (2019) as these researchers did not observe any significant morphological or volumetric associations with SES. However, our study differed in several methodological ways from McDermott et al., who measured SES using a two‐factor index of parental education and occupation, which are prestige‐based measures, whereas we used IPR, which is a resource‐based measure. McDermott et al. also had a much wider age range than the present study, including individuals aged 5–25 years. Therefore, ours is one of the first studies to show an association between SES and subcortical morphology in the basal ganglia in adolescents. Future studies are needed to replicate our findings.

We also observed a relationship between IPR and thalamic shape. Lower IPR was associated with outward thalamic curvature in males but inward thalamic curvature in females. Supplemental analyses of volume found a similar pattern of a positive association between IPR and thalamic volume in males and a negative association in females, although only for females did the result survive stringent Bonferroni correction. Our finding in females supports McDermott et al. (2019) who found that lower SES was associated with lower thalamic volume and inward shape changes. The thalamus is a key center for integrating basal ganglia networks that underlie the ability to modulate behaviors, for example, the expression of goal directed behaviors through movement, and the processes of emotion, motivation, and cognition that lead to movement (Haber & Calzavara, 2009). The thalamus is the final link from the basal ganglia back to the cortex, and it sends massive projections back to the striatum, and regulates cortical ensembles of neurons (Haber & Calzavara, 2009). The thalamus is also part of a fear circuit, for example, the paraventricular nucleus is activated by both physical and psychological stressors and plays a crucial role in inhibiting the amygdala (Penzo et al., 2015). Associations between thalamic structure and IPR in the present study could reflect a difficulty of low‐SES youth to recruit this region for the coordination of behavioral responses to stressful stimulation, and mobilization towards rewarding stimuli.

Mirroring earlier reports (Hair et al., 2015; Hanson et al., 2011; Noble et al., 2012, 2015), supplemental analyses did not indicate reliable associations between IPR and amygdala structure in volumetric analyses, after Bonferroni correction for multiple comparisons. Surface analysis in males showed some positive association in the right posterior amygdala, but there were no significant associations between IPR and the amygdala in females. A handful of studies have found smaller volumes among lower‐SES youth (Hanson et al., 2015; Luby et al., 2013; McDermott et al., 2019), but most do not, and it remains unclear what accounts for the differences. As with the hippocampus, discussed in the following section, BDNF could account for the positive associations between SES and amygdala curvature in males as abnormalities in BDNF methylation and mRNA expression in the amygdala are associated with early life adversity (Miskolczi et al., 2019).

Consistent with previous findings (Hanson et al., 2011, 2015; Leonard et al., 2015; Luby et al., 2013; McDermott et al., 2019; Noble et al., 2012), we observed indications of variations in hippocampal structure by IPR among males. However, unlike previous results, we observed that lower IPR males showed evidence of outward local shape variation in the right anterior hippocampus (CA1 region). These findings are in contrast to previous studies which tend to find reduced hippocampal volume to be associated with low SES. This finding is also inconsistent with the animal literature where early life stress reduces hippocampal volume (Seckl & Meaney, 2004), and density of glucocorticoid receptor expression, effects which attenuate the feedback sensitivity of the HPA axis. Our our findings of outward curvature associated with SES could potentially be explained by increased levels of BDNF in males in the present sample, as greater hippocampal volume has been associated with greater BDNF serum levels (Erickson et al., 2011). Further, dietary restriction (fasting) has been reported to enhance hippocampal neurogenesis in mice, an effect which is enhanced by BDNF (Lee, Duan, & Mattson, 2002), and low SES is associated with poor diet. However, in the absence of serum BDNF data, this hypothesis is speculative, and in need of future studies to explicitly test it.

### 4.2 Implications

Youths from low‐SES families experience more adverse outcomes across domains ranging from cognitive development, academic achievement, and mental and physical health (Duncan & Murname, 2011; Green et al., 2010; Matthews & Gallo, 2011; Miller et al., 2011; Noble et al., 2007; Reiss, 2013). These effects can persist across the life‐course (Hertzman & Boyce, 2010). Adolescence is a time of particular vulnerability to effects of environmental stress, as the brain experiences a period of prolonged plasticity (Tottenham & Galvan, 2016). Our findings in basal ganglia reward regions are consistent with the functional neuroimaging phenotype of children from lower SES families. For example, lower neighborhood quality predicts higher sensitivity to reward in the nucleus accumbens, caudate, putamen and the thalamus in adolescents (Gonzalez, Allen, & Coan, 2016). The reduced regulation of reward regions and stress‐induced remodeling of neural circuitry are hypothesized mechanisms underlying psychiatric (McEwen, 2008; Phillips, Drevets, Rauch, & Lane, 2003) and physical health problems (Nusslock & Miller, 2016).

### 4.3 Limitations

This study has limitations. The cross‐sectional design precludes conclusions about the timeline influence of SES on subcortical maturation. Future longitudinal research, which is currently lacking, is necessary to elucidate these relationships. Also, we chose to focus on IPR, however, SES is a multidimensional construct that also encompasses education, wealth, status, power, and knowledge. Although these dimensions are correlated, they have separable influences on children's development. Our LDDMM atlas was based on a single adult subject, future studies should consider an adolescent group‐based atlas. We note, however that all analyses passed visual quality inspection, and that the local shape variation for each participant was calculated from the population average of all participants in our study. Our results are seemingly in contrast to theories of accelerated maturation in the context of stress (Callaghan & Tottenham, 2016). However, a recent study found that while early life stress (0–5 years) was associated with accelerated gray matter development, ongoing social stressors in adolescence (14–17 years) were associated with delayed gray matter maturation, consistent with our findings (Tyborowska et al., 2018). Our study did not include measures of intelligence or cognition, therefore we could not examine their role in the observed associations, if any. We also could not directly compare males and females, because at the age of this sample, pubertal stage patterns strongly by sex. Finally, although we attempted to disentangle SES from racial and ethnic background, lower income minorities in the United States experience material hardship, psychosocial stress, and environmental pollution at disproportionately high rates. In a sample like ours, a portion of the variance associated with these pathways would be adjusted away in any model that includes both race and SES, which is why we did not covary race in the main analysis. A larger sample size would allow analyses of SES gradients separately within racial and ethnic groups.

## 5 CONCLUSIONS

Our study helps to clarify the association between SES and subcortical brain development in youth. It reveals unrecognized disparities in the structure of the thalamus, caudate, pallidum, putamen, and nucleus accumbens. These observations are consistent with models that assign SES a broad role in shaping development of threat and reward circuitries. Future research is needed to identify the specific exposures that underlie these associations. The quality of early caregiving is a potential candidate (McLaughlin, Sheridan, & Nelson, 2017). With additional research on the developmental persistence and behavioral implications of these patterns, we will acquire a more thorough understanding of the mechanisms through which socioeconomic conditions affect children's life outcomes.

## CONFLICT OF INTEREST

The authors declare no competing financial interests.

## Supporting information

Supporting Information

## Data Availability

We will make the dataset available upon reasonable request.
