# Supplementary material for: Subcortical structural variations associated with low socioeconomic status in adolescents
Source: Hum Brain Mapp. 2019 Oct 1;41(1):162–71. doi: 10.1002/hbm.24796 (PMC7268024; doi:10.1002/hbm.24796)
Supplement: Supplementary file 1 — Supporting Information [file HBM-41-162-s001.docx]

**Supplementary material for**

Jenkins, L. M.^1^, Chiang, J. J.^2^, Vause, K.^2^, Hoffer, L.^2^, Alpert, K.^1^, Parrish, T. B.^3^,

Wang, L.^1^, Miller, G.E.^2*^

*Subcortical structural variations associated with low socioeconomic status in adolescents*

^1^Department of Psychiatry and Behavioral Sciences, Northwestern University, Chicago, IL, USA

^2^Institute for Policy Research and Department of Psychology, Northwestern University, Chicago, IL, USA

^3^Departments of Radiology and Biomedical Engineering, Northwestern University, Chicago, IL, USA

**Contents**

**Table S1.** Subcortical volumes (mm^3^) by structure and hemisphere (page 3)

**Table S2.** Subcortical volumes multiple linear regression results for Males (pages 4-5)

**Table S3.** Subcortical volumes multiple linear regression results for Females (pages 6-7)

**Figure S1.** ROIs from Figures 2 (left, males) and 3 (right, females), combined as they appear in situ (page 8)

**Figure S2.** Regression of local shape variation onto Income:Poverty ratio for males, with race (Caucasian Y/N) and ethnicity (Hispanic Y/N), age, puberty category and intracranial volume as covariates and discussion (pages 9-10)

**Figure S3.** Regression of local shape variation onto Income:Poverty ratio for males, with race (Caucasian Y/N) and ethnicity (Hispanic Y/N), age, puberty category and intracranial volume as covariates (page 11) and discussion.

**Figure S4.** Regression of local shape variation onto Income:Poverty ratio for females, with race (Caucasian Y/N), ethnicity (Hispanic Y/N), age, puberty category and intracranial volume as covariates (page 12) and discussion.

**Table S1. Subcortical volumes (mm^3^) by structure**

|  | **Males** | | **Females** | |
| --- | --- | --- | --- | --- |
| **Structure** | **Mean volume** | **SD** | **Mean volume** | **SD** |
| amygdala | 2873.49 | 264.67 | 2603.63 | 266.12 |
| hippocampus | 5360.79 | 471.32 | 4975.48 | 478.88 |
| caudate | 7547.52 | 825.17 | 7049.06 | 715.72 |
| putamen | 10369.96 | 926.58 | 9584.42 | 830.44 |
| pallidum | 3766.08 | 369.33 | 3453.13 | 337.82 |
| nucleus accumbens | 820.47 | 88.40 | 752.54 | 85.94 |
| thalamus | 15209.04 | 1182.23 | 14117.03 | 1031.86 |

**Table S2. Multiple linear regressions of volume: Results for Males**

| **Structure** | **R^2^** | **F** | **p** | **IPR B** | **IPR SE (B)** | **IPR β** | **IPR p** |
| --- | --- | --- | --- | --- | --- | --- | --- |
| Hippocampus | .46 | 19.38 | <.001 |  |  |  |  |
| IPR |  |  |  | 20.76 | 9.35 | 0.18 | .029* |
| Age |  |  |  | -64.62 | 78.81 | -0.07 | .414 |
| Puberty |  |  |  | 0.22 | 59.13 | <0.01 | .997 |
| ICV |  |  |  | <0.01 | <0.01 | 0.59 | <.001*** |
| Amygdala | .41 | 15.49 | <.001 |  |  |  |  |
| IPR |  |  |  | 11.284 | 5.51 | 0.18 | .043 |
| Age |  |  |  | -5.956 | 46.451 | -0.01 | .898 |
| Puberty |  |  |  | -11.542 | 34.855 | -0.03 | .741 |
| ICV |  |  |  | <0.01 | <0.01 | 0.56 | <.001*** |
| Caudate | .52 | 24.54 | <.001 |  |  |  |  |
| IPR |  |  |  | 16.33 | 15.45 | 0.08 | .293 |
| Age |  |  |  | -106.78 | 130.22 | -0.06 | .414 |
| Puberty |  |  |  | -46.94 | 97.71 | -0.04 | .632 |
| ICV |  |  |  | <0.01 | <0.01 | 0.69 | <.001*** |
| NAcc | .47 | 20.01 | <.001 |  |  |  |  |
| IPR |  |  |  | 0.58 | 1.74 | 0.03 | .739 |
| Age |  |  |  | -17.05 | 14.67 | -0.10 | .248 |
| Puberty |  |  |  | -9.18 | 11.01 | -0.07 | .407 |
| ICV |  |  |  | <0.01 | <0.01 | 0.67 | <.001*** |
| Pallidum | .41 | 15.46 | <.001 |  |  |  |  |
| IPR |  |  |  | -5.78 | 7.69 | -0.06 | .455 |
| Age |  |  |  | 9.51 | 64.84 | 0.01 | .884 |
| Puberty |  |  |  | -24.08 | 48.65 | -0.04 | .622 |
| ICV |  |  |  | <0.01 | <0.01 | 0.66 | <.001*** |
| Putamen | .35 | 12.44 | <.001 |  |  |  |  |
| IPR |  |  |  | 12.29 | 20.11 | 0.06 | .543 |
| Age |  |  |  | -87.24 | 169.50 | -0.05 | .608 |
| Puberty |  |  |  | -27.24 | 127.18 | -0.02 | .831 |
| ICV |  |  |  | <0.01 | <0.01 | 0.57 | <.001*** |
| Thalamus | .61 | 35.38 | <.001 |  |  |  |  |
| IPR |  |  |  | 40.75 | 19.96 | 0.14 | .044* |
| Age |  |  |  | 37.00 | 168.28 | 0.02 | .826 |
| Puberty |  |  |  | -195.40 | 126.27 | -0.11 | .125 |
| ICV |  |  |  | 0.01 | <0.01 | 0.74 | <.001*** |

*Note.* **p*< .05, ***p*< .01, ****p*< .001. † significant at Bonferroni adjusted p< .007.**Table S3. Multiple linear regressions of volume: Results for Females**

| **Structure** | **R^2^** | **F** | **p** | **IPR B** | **IPR SE (B)** | **IPR β** | **IPR p** |
| --- | --- | --- | --- | --- | --- | --- | --- |
| Hippocampus | .44 | 30.36 | <.001 |  |  |  |  |
| IPR |  |  |  | -4.08 | 10.74 | -0.02 | .704 |
| Age |  |  |  | -35.64 | 54.34 | -0.04 | .513 |
| Puberty |  |  |  | -26.14 | 51.29 | -0.03 | .611 |
| ICV |  |  |  | <0.01 | <0.01 | 0.67 | <.001*** |
| Amygdala | .40 | 25.37 | <.001 |  |  |  |  |
| IPR |  |  |  | -0.72 | 6.19 | -0.01 | .908 |
| Age |  |  |  | -34.24 | 31.35 | -0.07 | .276 |
| Puberty |  |  |  | 5.96 | 29.59 | 0.01 | .841 |
| ICV |  |  |  | <0.01 | <0.01 | 0.63 | <.001*** |
| Caudate | .48 | 35.31 | <.001 |  |  |  |  |
| IPR |  |  |  | -44.01 | 15.50 | -0.17 | .005** † |
| Age |  |  |  | -121.58 | 78.46 | -0.10 | .123 |
| Puberty |  |  |  | -89.77 | 74.05 | -0.07 | .227 |
| ICV |  |  |  | <0.01 | <0.01 | 0.70 | <.001*** |
| NAcc | .39 | 24.79 | <.001 |  |  |  |  |
| IPR |  |  |  | -5.30 | 2.01 | -0.17 | .009 |
| Age |  |  |  | -13.53 | 10.17 | -0.09 | .185 |
| Puberty |  |  |  | -10.04 | 9.60 | -0.07 | .297 |
| ICV |  |  |  | <0.01 | <0.01 | 0.63 | <.001*** |
| Pallidum | .55 | 47.32 | <.001 |  |  |  |  |
| IPR |  |  |  | -20.08 | 6.79 | -0.17 | .004** † |
| Age |  |  |  | -47.81 | 34.35 | -0.08 | .166 |
| Puberty |  |  |  | -28.63 | 32.42 | -0.05 | .379 |
| ICV |  |  |  | <0.01 | <0.01 | 0.76 | <.001*** |
| Putamen | .47 | 34.30 | <.001 |  |  |  |  |
| IPR |  |  |  | -53.78 | 18.11 | -0.18 | .003** † |
| Age |  |  |  | -101.98 | 91.66 | -0.07 | .268 |
| Puberty |  |  |  | -94.02 | 86.52 | -0.07 | .279 |
| ICV |  |  |  | 0.01 | <0.01 | 0.70 | <.001*** |
| Thalamus | .59 | 56.21 | <.001 |  |  |  |  |
| IPR |  |  |  | -55.31 | 19.73 | -0.15 | .006** † |
| Age |  |  |  | -55.18 | 99.89 | -0.03 | .581 |
| Puberty |  |  |  | -66.99 | 94.28 | -0.04 | .478 |
| ICV |  |  |  | 0.01 | <0.01 | 0.79 | <.001*** |

*Note.* **p*< .05, ***p*< .01, ****p*< .001, † significant at Bonferroni adjusted p< .007.


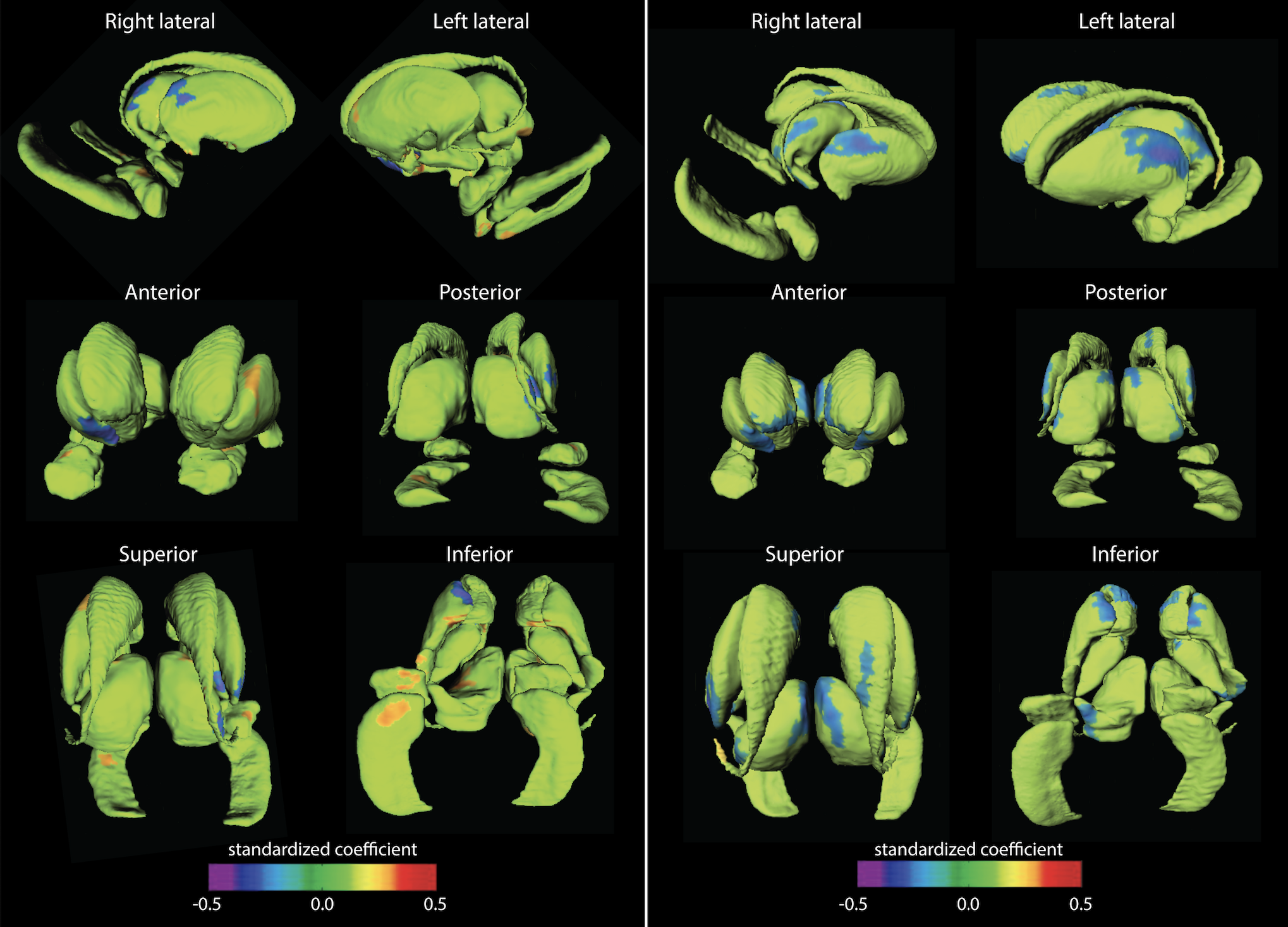


*Figure S1. ROIs from Figure 1 (left, males, right, females), combined as they appear in situ. Vertex-wise RFT corrected FWER cluster threshold p<.01, FWER p< .007, Bonferroni adjusted.*


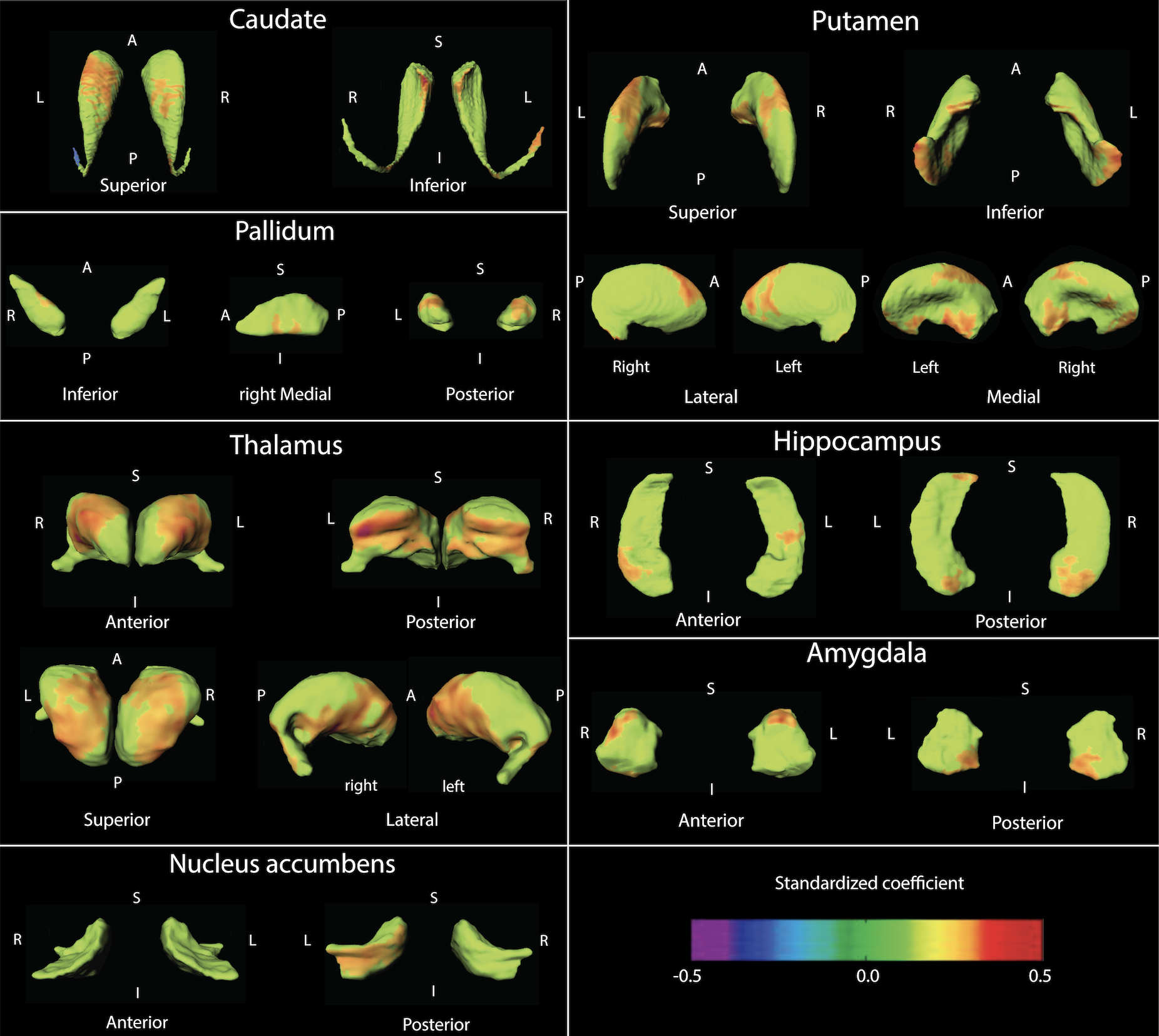


Figure S2. Regression of local shape variation onto Income:Poverty ratio for males, with age and puberty category as covariates. Vertex-wise RFT corrected FWER cluster threshold *p*<.01, FWER *p*< .007 per ROI, Bonferroni adjusted. In regions with warmer colours, lower IPR is related to more outward local shape variation. In regions with cooler colours, lower IPR is related to more inward local shape variation.

*Note*. A=anterior, I=inferior, P=posterior, S=superior, L=left, R=right.

Comparison between Figure 1 and Figure S2 reveals that removing ICV as a covariate results in more extensive positive associations between IPR and shape variation in males, in all ROIs. This includes the nucleus accumbens which did not show any significant associations when ICV was covaried, and the pallidum which showed negative associations when ICV was covaried and positive when it was not. For females, when ICV was not covaried, there were no longer any significant associations between IPR and shape variation. This finding and the stronger results observed in Figure S2 than Figure 1 are not surprising given the correlations between IPR and ICV which are .21 for females and .31 for males. Considering the IPR coefficients in Figure 1 of around +0.2 for males and -0.2 for females, and the correlations between IPR and ICV just stated, it stands to reason that when the unique variance accounted for by ICV is no longer statistically controlled for, the coefficients become larger for males, as positive coefficients of IPR plus positive coefficients of ICV results in larger positive coefficients. However, for females, who have negative associations between IPR in Figure 1, when positive coefficients of ICV are added to these, the coefficients equate to around zero, hence no significant associations.


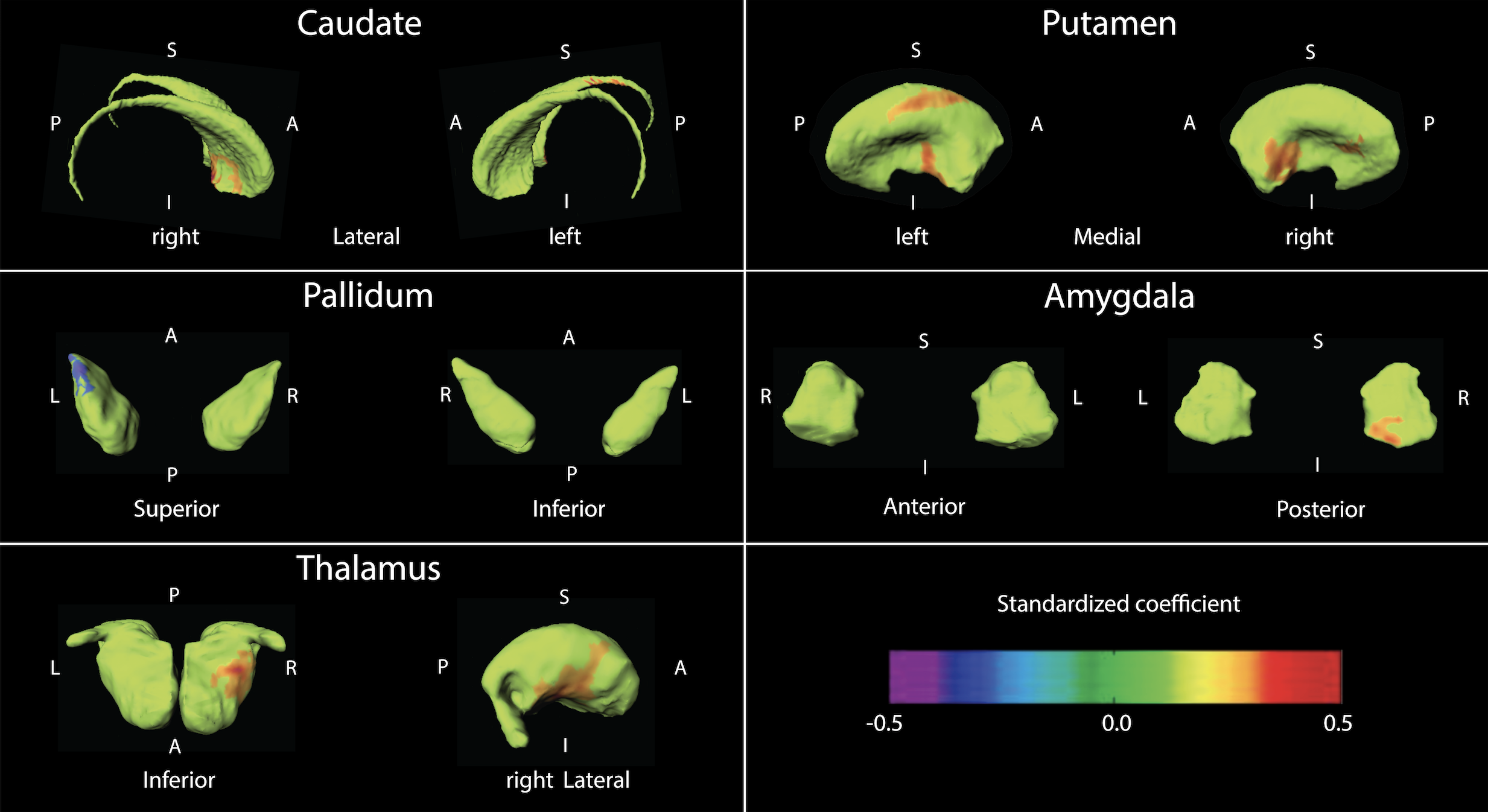


Figure S3. Regression of local shape variation onto Income:Poverty ratio for males, with race (Caucasian Y/N), ethnicity (Hispanic Y/N), age, puberty category and intracranial volume as covariates. Vertex-wise RFT corrected FWER cluster threshold *p*<.01, FWER *p*< .007 per ROI, Bonferroni adjusted. In regions with warmer colours, lower IPR is related to more outward local shape variation. In regions with cooler colours, lower IPR is related to more inward local shape variation.

*Note*. A=anterior, I=inferior, P=posterior, S=superior, L=left, R=right.

For the surface analysis of males, a comparison of Figures 1 and S3 reveals that the majority of clusters remained significantly associated with IPR when adding race and ethnicity as covariates. These included the positive coefficient clusters in the amygdala, caudate tail, medial putamen, and right lateral thalamus, and a negative coefficient cluster in the left pallidum. However, some associations were no longer significant when race and ethnicity were added as covariates, including the positive coefficients in the right anterior hippocampus, left lateral and posterior thalamus and left lateral caudate head

.


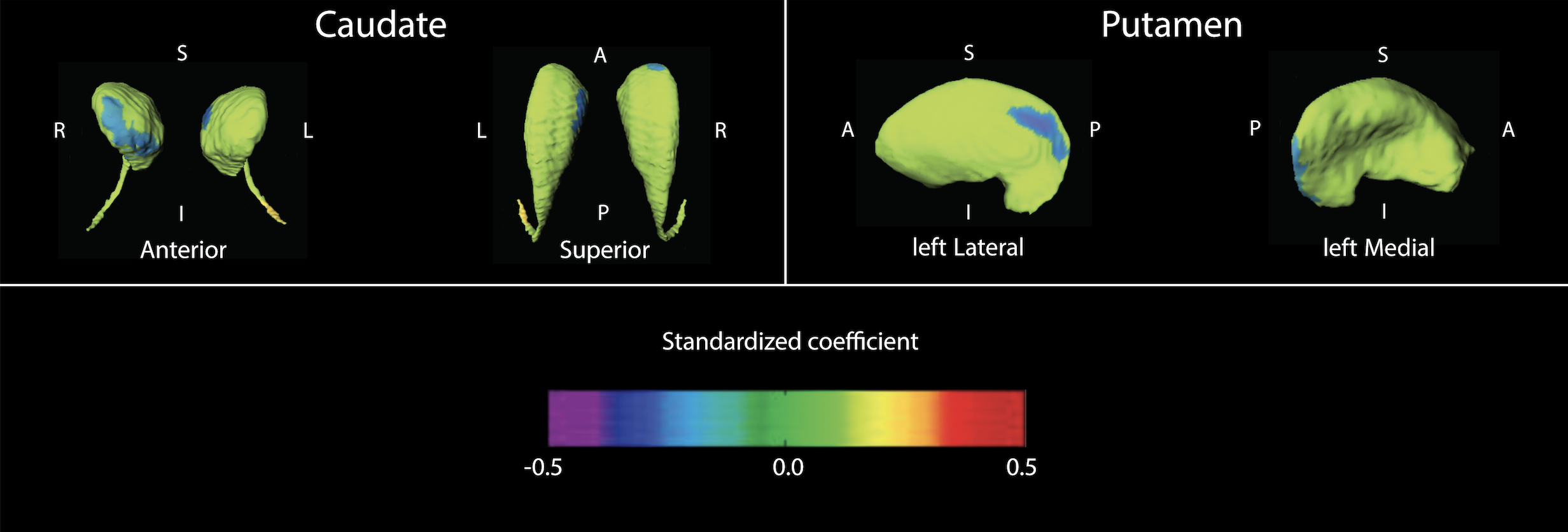


Figure S4. Regression of local shape variation onto Income:Poverty ratio for females, with race (Caucasian Y/N), ethnicity (Hispanic Y/N), age, puberty category and intracranial volume as covariates. Vertex-wise RFT corrected FWER cluster threshold *p*<.01, FWER *p*< .007 per ROI, Bonferroni adjusted. In regions with warmer colours, lower IPR is related to more outward local shape variation. In regions with cooler colours, lower IPR is related to more inward local shape variation.

*Note*. A=anterior, I=inferior, P=posterior, S=superior, L=left, R=right.

For the surface analysis of females, comparison between Figure 1 and Figure S4 shows that when race and ethnicity were added as covariates, the negative coefficients in the thalamus and pallidum were no longer significant. However, the negative clusters in the caudate and the left lateral putamen remained similar.
